# Distribution, speciation, and assessment of heavy metals in sediments from Wadi Asal, Red Sea, Egypt

**DOI:** 10.1007/s10661-024-12363-1

**Published:** 2024-01-30

**Authors:** Amal Mosalem, Mostafa Redwan, Ahmed A. Abdel Moneim, Shaymaa Rizk

**Affiliations:** Geology Department, Faculty of Science, Sohag University, Sohag, 82524 Egypt

**Keywords:** Heavy metals, Sediment contamination, Sequential extraction, Mining activities, Wadi Asal of Egypt

## Abstract

Globally, the environmental contamination of stream sediments due to geogenic and anthropogenic sources is of growing concern. In this study, the heavy metals (Cd, Co, Cr, Cu, Ni, Pb, and Zn) in 22 superficial sediments in Wadi Asal, Red Sea, Egypt, were explored to assess sediment sources, the mobility of chemical species, and the degree of contamination in sediments. Therefore, the total heavy metal values in the fine fraction (< 63 μm), a five-step sequential extraction on selective samples, risk assessment, and principal component analysis (PCA) were applied. The mobility of heavy metals in Wadi Asal sediments, according to non-residual fraction percent, declines in the following order: Cd (90.9%) > Pb (85.2%) > Co (84.4%) > Cu (80.8%) > Zn (75.9%) > Ni (48.4%) > Cr (39.6%); indicating the high mobility of Cd, Zn, Pb, Cu, and Co. The mean metal contamination factor (CF) order is Cd (10.96) > Ni (3.91) > Cr (2.77) > Zn (2.18) > Pb (2.10) > Co (1.12) > Cu (0.70). The Geo-accumulation Index (I_geo_) is decreased in the following order: Cd (2.19) > Ni (0.78) > Cr (0.55) > Zn (0.44) > Pb (0.42) > Co (0.22) > Cu (0.14). The risk assessment code (RAC) revealed very high to high risk for Cd, Co, and Pb. The results pointed out that the metals Cr, Co, Cu, and Ni are from geogenic sources, while Zn, Cd, and Pb are from anthropogenic sources due to Pb–Zn mining activities. Based on the threshold effect level (TEL), Cd, Cr, Ni, and Pb have adverse effects on living organisms. According to these findings, the area along Wadi Asal and the downstream regions on the beach are highly polluted and heavy metal monitoring in sediments and aquatic organisms is recommended.

## Introduction

Heavy metals are of vital ecological importance as a result of their toxicity, degradation resistance, and tendency to bioaccumulate (Diagomanolin et al., 2004). Many areas affected by high contamination loads are visible over short distances in waterways and flood basins around the world (Byrne et al., 2010). The fate of metal drift along the river bank depends on chemical interactions and erosion rates (Pulford et al., 2009). Flooding is among the most expensive natural catastrophes that result in destruction and fatalities (Alexander, 1993; Prama et al., 2020). To quantify the environmental disturbance related to elemental distribution, researchers should quantify metal values in the ecosystem close to inactive mines and smelting locations and the agents controlling their dispersion. Chemical fractionation of heavy metal content in sediments becomes necessary to reveal their prospective risks affecting plants, animals, and human health. Determining the different heavy metal fractions by utilizing several sequential extractions is the key to understanding the geochemical processes and bioavailability. Sequential extraction is used to obtain comprehensive data on the mechanisms of occurrence, origin, biological and physicochemical availability, and potential trace metal mobilization (Li et al., 2024; Tessier et al., 1979). Heavy metals show different trends in potential toxicity, transfer, and availability where heavy metals are located in contrasting chemical forms in sediments (Li et al., 2000). The spatial distribution of heavy metals is crucial for risk analysis and management of contaminated sites (Man et al., 2022).

In Egypt, eight lead and zinc mineralizations (Zog El Bohar, Asal, Wizr, Um Gheig, Abu Ghorban, Abu Anz, Gebel El Rusas, and Ranga) are disseminated in the Miocene sediments on the Red Sea coastal zone. Over the past three decades, the Egyptian government has given much thought to expanding the Red Sea coast into a promising area for economic development, especially tourism, mining, and agricultural activities. Due to the rapid expansion of these activities in the Safaga-Quseir coastal area, sustainable development and environmental management and protection approaches should be considered. This study’s main aim is to identify the possible effects of Pb–Zn mining activities on heavy metal speciation in sediments. The specific aims are to (1) quantify the concentration of heavy metals in beach sediments and along the wadis, (2) identify sediment sources, (3) categorize the heavy metals into definite groups using multivariate statistical methods, and (4) evaluate contamination degree, risk assessment, and enrichment in sediments.

### The study area characteristics

**Fig. 1:**
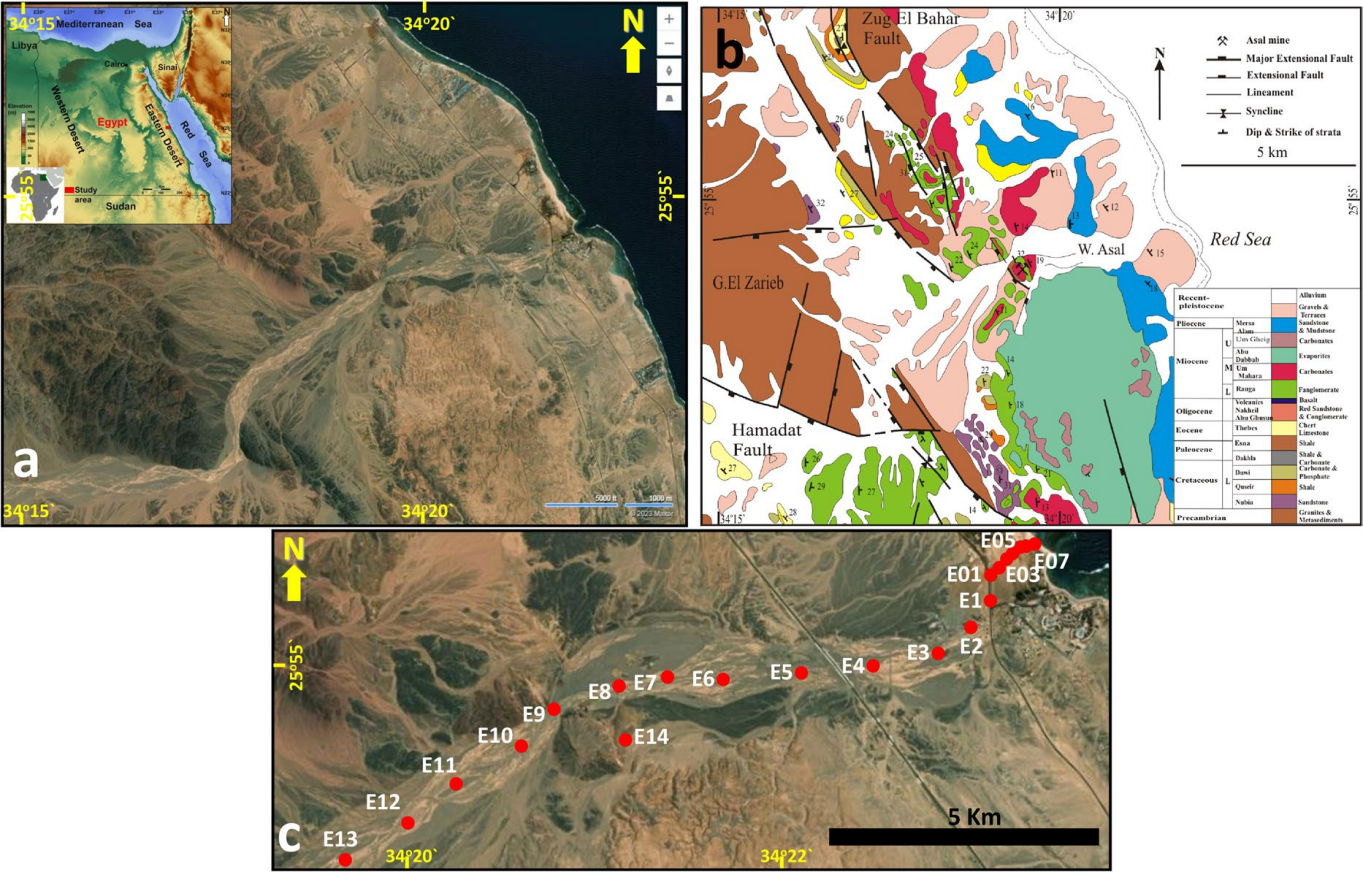
Location (**a**), detailed geologic (**b**) (modified after Khalil & McClay, 2009) and sampling sites maps of the Wadi Asal area

Wadi Asal basin lies 22 km south of El Quseir and is bounded by latitudes 25°35′26.65″ and 25°59′4.11″N and longitudes 34°00′07.6″ and 34°23′32.16″E. The Asal mine is located about 5 km west of Quseir-Mersa Alam old road (Fig. 1). The stratigraphy of NW Red Sea coastal sediments is divided into pre-rift and syn-rift sequences. The pre-rift sequence consists of a Pre-Cambrian crystalline basement at the base, followed upward by Mesozoic-Cenozoic strata, while the syn-rift strata were formed during the Oligocene to recent times. The Pb–Zn mineralization source is sedimentary/hydrothermal genesis (El Aref & Amstutz, 1983). The origin of Pb and Zn in conglomerates of carbonate-hosted deposits and hydrothermal solutions comes from the Pre-Cambrian basement. These ore deposits are found in the mixed clastic and carbonate facies of Um Mahara Formation (El Aref & Amstutz, 1983). The sandstone is separated from the top calcareous rocks by an erosional surface covered by a reworked polymictic conglomerate layer. Galena exhibits typical stratiform and stratabound patterns, forming layers or bedding structures symmetrical and congruent to the bedding structures of sandstone country rock and disseminated cementative concretions. Galena indicates close symmetry and congruency with the enclosing sandstone, which denote a sedimentary syndiagenetic origin. The heavy mineral concentrations in the sandstone include martitized magnetite, hematite, zircon, rutile, monazite, pyrite, scheelite, and gold, with cement of pyrolusite and manganite. The clay country rock plays a principal part in the separation and uptake of zinc. The Fe oxy-hydroxides and sulfides take a notable part in trapping Pb ions (Abou-El-Anwar & Mekky, 2013).

## Materials and methods

### Geochemical studies

#### Sampling

Twenty-two surface sediments (from the top 0–10 cm) were collected from Wadi Asal for heavy metal quantifications (Cd, Co, Cr, Cu, Ni, Pb, and Zn), with about 300 m of consecutive distance between samples. The geographic distribution of the sample sites was indicated by Garmin DAKOTA 10 GPS device (Fig. 1c). The samples were stored in plastic bags until analysis.

#### Geochemical analysis

The total digestion method was applied to the bulk sample to determine the heavy metal total concentrations following the Environmental Quality Control Standard for soil contamination (Mason, 1992). The sediment samples were air-dried, then passed through the 63-μm sieve and totally digested in a mixture of strong acids (4 ml HNO_3_ 69%, 4 ml HClO_3_ 40%, and 15 ml HF 40%) by microwave (method 3051A; Kouadia & Trefry, 1987) until incipient dryness. The sequential extraction procedure of Tessier et al. (1979) was carried out to identify the different chemical forms in sediments and assess their risk according to their mobility and bioavailability. The speciation was applied to seven selective samples. The reagents and conditions employed in the extraction procedure of the different fractions are F1 exchangeable (using 1 M NaOAc (pH = 8.2), at 25 °C for 1 h); F2 carbonates (using 1 M NaOAc and buffer pH = 5 with HOAc, at 25 °C for 5 h); F3 Fe–Mn oxides (using 0.04 M NH_2_OH.HCl in 25% (v/v) HOAc at 96 ± 3 °C); and F4 organic matter (using 0.02 M HNO_3_ and 30% H_2_O_2_ and buffer pH = 2 with HNO_3_ at 85 ± 2 °C for 2 h. A second was added to 30% H_2_O_2_ (pH 2 with HNO_3_) and heated again to 85 ± 2 °C for 3 h. After cooling, 3.2 M NH_4_OAc in 20% (v/v) HNO_3_ added and diluted to 20 ml and agitated for 30 min); F5 residual (using HF—HClO_3_ (5:1), HF—HClO_3_ (10:1), HClO_3_—HCL (12N) and diluted to 25 ml). Details of the sequential extraction procedure were reported by Li et al., (1995a & b). Concentrations of the investigated metals (Cd, Co, Cr, Cu, Ni, Pb, and Zn) were quantified in different aliquots by an Atomic Absorption Spectrophotometer (Perkin Elmer, A. Analyst 400).

#### Statistical and multivariate analyses

Descriptive statistics were applied to calculate the mean, standard deviation, minimum, maximum, median, and principal component analysis (PCA) by Statistica 13 software. It interprets the origins, the relationships between heavy metals in sediment samples, the geochemical behavior within the different fractions, the similarity and dissimilarity of variables, inter-variable relationship, mobility, and the correlation coefficient for the estimated different chemical elements and heavy metals via extracting a reduced number of latent factors (principal components, PCs) (Loska & Wiechuła, 2003).

#### Heavy metals risk assessment

##### Contamination factor (CF)

CF is used to determine the intensity of anthropogenic contamination in the sediments (Hakanson, 1980) by comparing heavy metal concentrations in soil samples in the investigated area with the average world background value (Kabata-Pendias, 2011). The values of (CF) are calculated using the following formula:

$${\text{CF}}=\left({{\text{C}}}_{{\text{m}}}/{{\text{C}}}_{{\text{n}}}\right)$$

C_m_ is the heavy metal level, and C_n_ is the background level of each metal. CF can be classified into CF < 1 low, 1 ≤ CF ≤ 3 moderate, 3 ≤ CF ≤ 6 considerable, and 6 ≤ CF very high contaminations (Hakanson, 1980).

##### Geo-accumulation Index (I_geo_)

The geo-accumulation index (I_geo_) was originally defined by Müller (1969), in order to determine metal contamination in sediments, by comparing current values with pre-industrial levels. I_geo_ can be quantified by the following formula:

$${{\text{I}}}_{{\text{geo}}}={{\text{log}}}_{2}\left({{\text{C}}}_{{\text{m}}}/1.5{{\text{C}}}_{{\text{n}}}\right)$$

The 1.5 is a correction factor utilized to minimize background variation due to lithogenetic origins (Redwan & Elhaddad, 2022). The I_geo_ can be grouped into seven classes (≤ 0 unpolluted, ≤ 1 unpolluted to moderately polluted, ≤ 2 moderately polluted, ≤ 3 moderately to highly polluted, ≤ 4 highly polluted, ≤ 5 highly to very highly polluted, > 5 very highly polluted) (Ahmed et al., 2023).

##### Risk assessment code (RAC)

This index is identified as the ratio of the exchangeable ions (F1) and carbonate (F2) fractions to the total (Ct) fractions (speciation) of heavy metal. RAC is calculated using the following formula (Perin et al., 1985; Singh et al., 2005):

$${\text{RAC}}=\left({\text{F}}1+{\text{F}}2/{\text{Ct}}\right)\times 100$$

RAC grouped into five sets: RAC < 1 no, 1 ≤ RAC < 10 low, 10 ≤ RAC < 30 medium, 30 ≤ RAC < 50 high, and RAC ≥ 50 very high risks.

##### Quality control

To authenticate the procedure in this research, all the chemical compounds and reagents were of analytical grade and purchased from Merck (Darmstadt, Germany), unless otherwise stated. Extracting solutions were prepared from analytical grade chemicals utilizing ultrapure deionized water. The glassware and plastic materials utilized were earlier treated for 24 h in 10% (v/v) HNO_3_ and then rinsed with deionized water. Procedure blanks and quality control samples developed from standard solutions were used to ensure sample accuracy. The precision for the analysis of the standard solution was within 10%. Quality control of the achieved results was showed using of certified reference materials (CRM), blank reagents, and triplicate samples. Quality control for the total metal concentrations was conducted using standard sediment reference material from the National Research Council of Canada, namely PACS-2 certified reference material (CRM), in mg/g (Table 1). Table 1 reveals that all of the metals studied recover well (the measured concentrations were compared to the actual spike concentration added) (El Zokm et al., 2015). The metal recovery concentrations obtained in the certified standard ranged between 96 and 113.9%, which is considered acceptable.

**Table 1:** Measured and certified values of Zn, Pb, Cu, Cd, Ni, Co, and Cr concentrations (mg/kg) using standard certified reference material from National Research Council of Canada (CRM PACS 2)

PACS 2	Certificate value	Measured value	Recovery rate %
Zn	364	403.17	110.8
Pb	183	198.01	108.2
Cu	310	298	96.1
Cd	2.11	2.25	106.6
Ni	39.5	45	113.9
Co	11.5	13.1	113.9
Cr	90.7	96	105.8

## Results and discussion

**Fig. 2:**
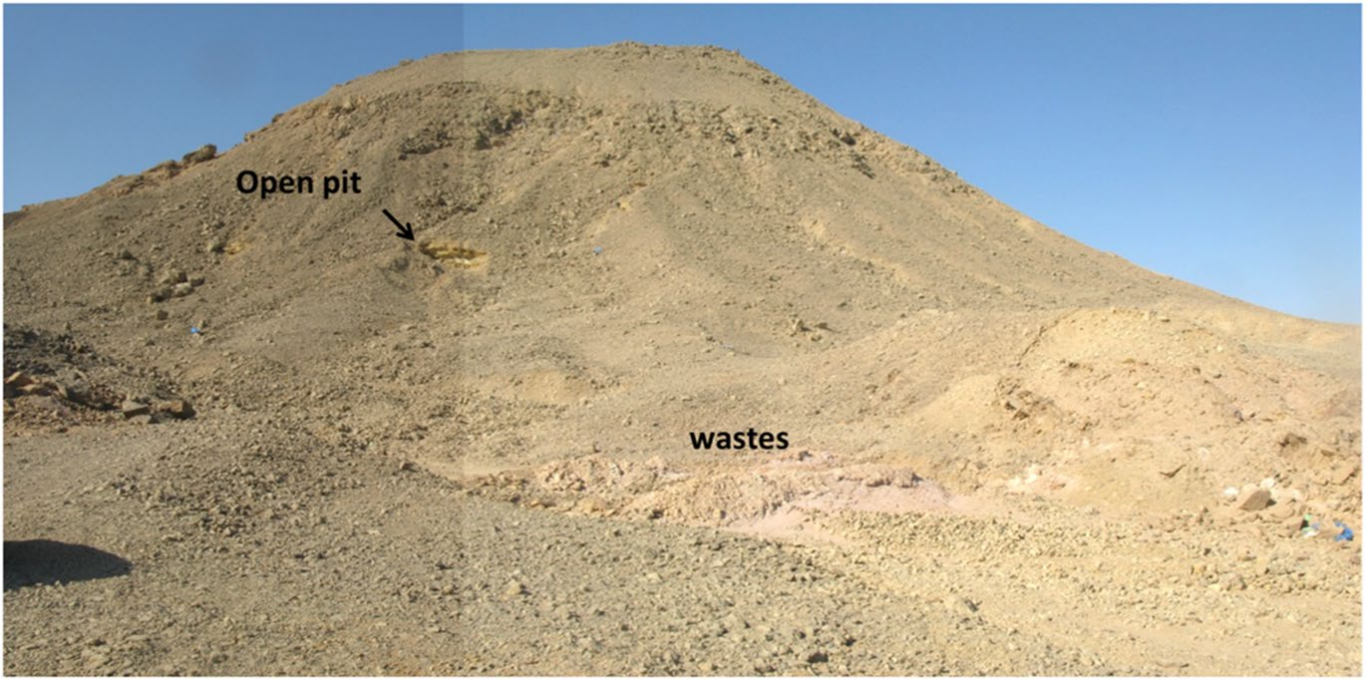
Asal mine area with Pb–Zn mineralization in Um Mahara Formation

Mining activities and later mine waste deposition at dumping locations have a substantial impact on the nearby environment. Based on the observations of El Aref & Amstutz (1983), the Asal ore deposits are located in mixed clastic and carbonate facies of the Um Mahara Formation (Fig. 1b). Stratiform and stratabound galena are found in layers or bedding structures. The minerals magnetite, hematite, zircon, rutile, monazite, pyrite, and scheelite are encountered in association with the ore (El Aref & Amstutz, 1983). Field observations revealed mine wastes disposed of on the downstream flow path (Fig. 2). This is typically the primary contamination source in Wadi Asal sediments, in addition to a few erosion materials from the enclosing hill slopes.

An extremely dry climate with little frequent precipitation characterizes Egypt’s Eastern Desert. In the study area, precipitation occasionally takes the form of short, powerful rainfall episodes. Most rainfalls are typically infrequent and occur in intense thunderstorms from November to April. According to El-Haddad (2020), the flood susceptibility map showed the study area is classified as a high-to-very-high-risk area. In turn, the erosion and leaching of the polluted floodplain sediments were recognized as important factors influencing environmental pollution proportional to the degree of historical pollution (Chen et al., 2014; Redwan & Rammlmair, 2017). Windblown and water-transported particles as well as dissolved matter from the mine site might reach the river bed.

### Descriptive statistics of total heavy metal concentrations in sediments

The heavy metal pollution in marine sediments, beaches, and seashells along the Red Sea coast was investigated using numerous authors (Abdelkader et al., 2018; Attia & Ghrefat, 2013; El-Metwally et al., 2019; El-Sorogy et al., 2012; Madkour, 2005; Madkour et al., 2012; Mansour et al., 2011; Philobbos et al., 1983; Ramadan & Zaid, 2013; Redwan & Rammlmair, 2017; Salem et al., 2014; Temraz & El-Sawey, 2017). The overall contamination came from lithologic nature and anthropogenic activities such as land filling, oil pollution, and utilizing antifouling and anticorrosive paints from the fishing and shipping processes of phosphate ore. The distribution of total heavy metal concentrations (Cd, Co, Cr, Cu, Ni, Pb, and Zn) fluctuated widely in Wadi Asal Basin, following the order: Cr > Zn > Ni > Pb > Cu > Co > Cd (Table 2). The concentrations of Cr in sediments varied from 49.75 to 463.35 mg/kg, followed by Zn which varied from 38.25 to 483.2 mg/kg. The concentrations of Ni in sediments varied from 48.85 to 276.7 mg/kg. The Pb concentrations varied from 30.6 to 328.6 mg/kg, followed by Cu which varied from 10.8 to 97.15 mg/kg. The concentrations of Co in sediments varied from 3.35 to 23.2 mg/kg, followed by Cd which varied from 3.20 to 9.90 mg/kg. The examined sediment samples show a high and extensive range of toxic heavy metal values due to the association of Cd with Zn–Pb mine activities. The descriptive statistics of metals are represented in Table 2. Zn and Pb display very high maximum values of 483.2 and 328.6 mg/kg with means of 152.7 and 56.6 mg/kg, respectively, due to the presence of Pb–Zn mineralization. Cd has a high maximum value of 9.9 mg/kg in the study area with a mean value of 4.495 mg/kg due to its association with Zn–Pb mineralization. The average concentration of Cd in sediments exceeded the concentration reported on the worldwide side of 0.41 mg/kg (Kabata-Pendias, 2011).

**Table 2:** Descriptive statistics of total concentration of heavy metals

	Min	Max	Mean	L.q	median	U.q	SD	CV	SK	Kur
Cd	3.20	9.90	4.495	3.75	4.18	4.65	1.41	31.40	3.01	10.59
Cr	49.75	463.35	164.73	99.55	122.10	250.5	108.13	65.64	1.385	1.295
Co	3.35	23.2	12.68	9.90	12.55	14.90	4.135	32.61	0.375	1.353
Cu	10.8	97.15	27.42	21.90	23.28	27.50	17.22	62.81	3.431	13.75
Pb	30.6	328.6	56.60	34.75	38.03	40.2	66.97	118.3	3.726	14.27
Ni	48.85	276.7	113.3	76.45	86.83	142.8	59.81	52.78	1.505	1.617
Zn	38.25	483.2	152.7	62.15	92.15	258.2	128.3	84.03	1.276	0.541

*Min*, minimum; *Max*, maximum; *L.q*, lower quartile; *U.q*, upper quartile; *SD*, standard Deviation; *CV*, Coef. variation; *SK*, skewness; *Kur*, kurtosis

Cr has an elevated maximum value of 463.35 mg/kg reflecting its natural sources owing to the chemical weathering of chromite minerals in basement rocks such as serpentinites, talc-carbonates, and metasediments (Harraz et al., 2012; Wedepohl, 1978). Ni has a very high maximum concentration of 276.7 mg/kg with an average of 113.3 mg/kg due to the presence of Pb–Zn mining activities. Co shows a wide range varying from 3.35 to 23.2 mg/kg related to weathering from the Pb–Zn mineralization in the country rocks and associated wastes (Fig. 2) (Lu et al., 2018; Redwan & Rammlmair, 2017) and close to the background values around the Egyptian basement complex. The cumulative accumulation of these heavy metals in the sediments is recorded as a result of the intensive Zn–Pb mine activities. The highest Cu, Zn, Cr, Pb, Cd, Co, and Ni levels in sediments increase their mobility. These values are several times higher than the background contents in the worldwide sediments (Kabata-Pendias, 2011). Furthermore, higher concentrations of heavy metals were also observed at both the midstream and downstream of Wadi Asal, which may exert potential influences of the surrounding Egyptian basement rocks and efflorescence salts.

### Aerial distribution maps of heavy metals

**Fig. 3:**
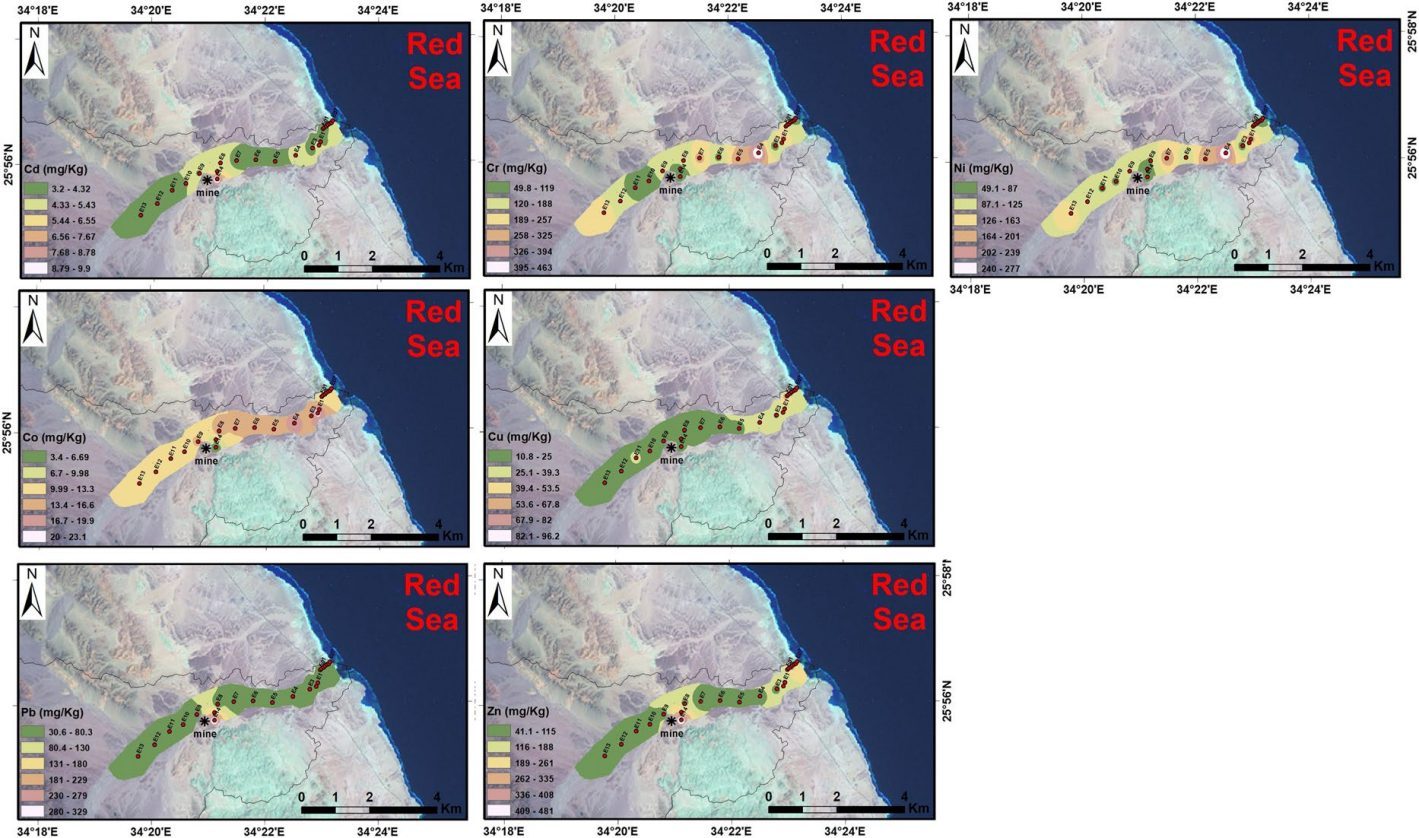
Aerial distribution maps of heavy metals along Wadi Asal

Aerial distribution maps (Fig. 3) exhibited high levels of Cd, Pb, and Zn near the mine areas in Wadi Asal. Cr and Co showed minor enrichment near the mine areas of Wadi Asal and an increase in some upstream parts (basement source rocks) and downstream sites (mobility). The moderate level of Cu in the estimated sediments is due to the occurrence of Egyptian basement complex rocks and Pb–Zn mining activities (Abdalla & Khalifa, 2013; Lu et al., 2018; Redwan & Rammlmair, 2017; Wedepohl, 1978). The high level of Ni Cd, Pb, and Zn in the study area, especially near the downstream areas of Wadi Asal, may be ascribed to the existence of Pb–Zn mining as well as the basic and ultrabasic rocks recognized at these central wadis of the Red Sea (El-Shater, 1985).

### Interrelationship of geochemical elements in sediments

**Fig. 4:**
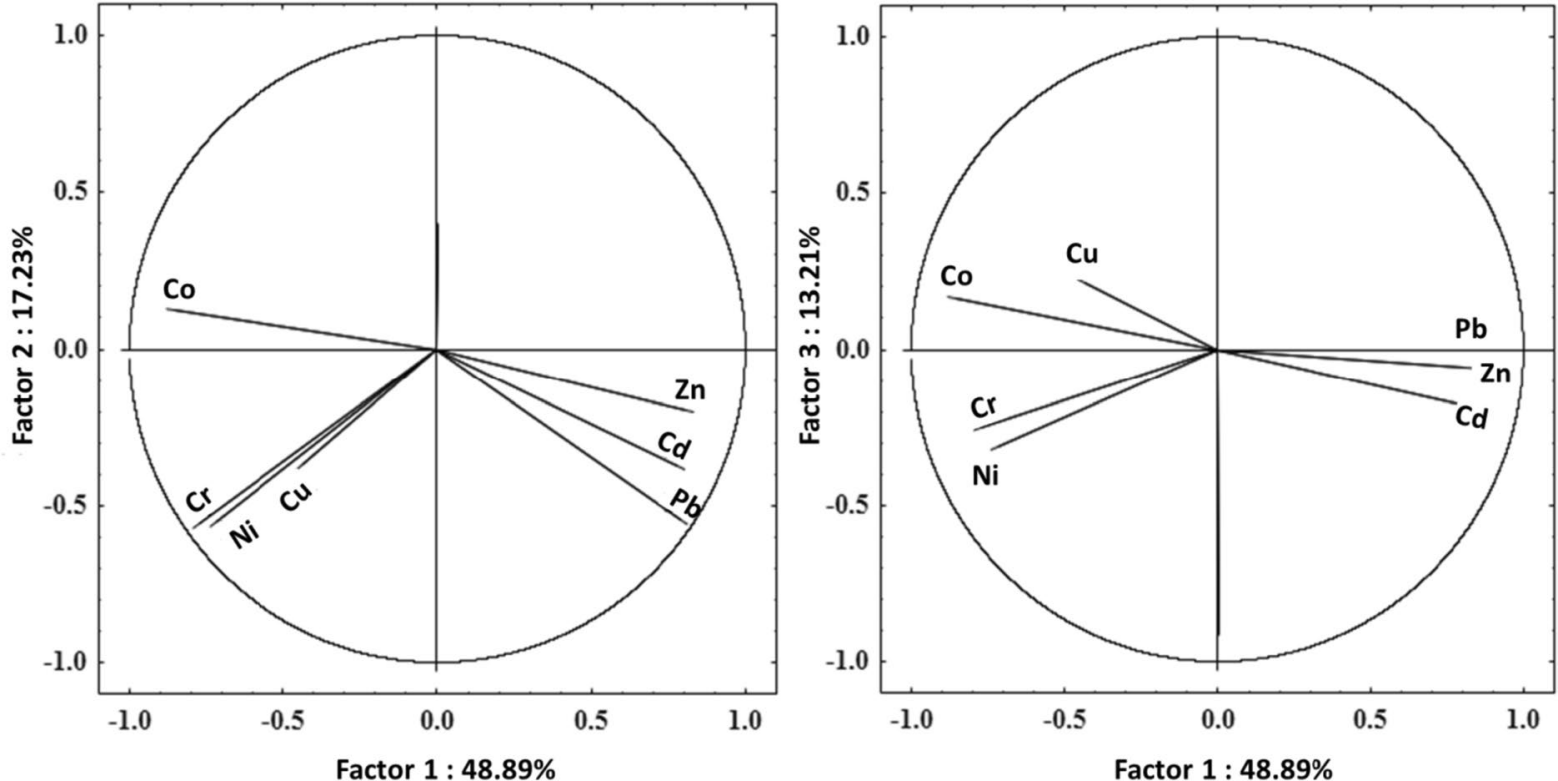
Loading the first, second, and third principal factors of heavy metals

Principal component analysis (PCA) was accomplished to quantify the hypothetical source of heavy metals (natural or human) in sediment samples. According to the Kaiser criterion (Kaiser, 1960), three principal components extracted from the variables with eigenvalues > 1 were retained for further analysis. The variances of the three principal components were explained by 79.33% of the total variance for this study. The relations between these metals based on the three principal components are exhibited in Fig. 4. Zn, Cd, and Pb (factor 1) which accounted for 48.89% of the total variance, have strong positive loading and relations (Zn and Cd *r* = 0.53), (Zn and Pb *r* = 0.68) and (Cd and Pb *r* = 0.83) (Table 3). Zn, Cd, and Pb are strongly correlated in the same group, indicating their similar distribution patterns and sources, referring to their anthropogenic sources from Pb–Zn mining activities as the primary source of contamination (Fig. 4). Cr, Ni, and Co (factor 2) have strong negative loading (accounted for 17.23% of the total variance) belong to one group that reflect the same geogenic sources from the chemical weathering of complex basement rocks such as serpentinites, talc-carbonates, and metasediments (Abdalla & Khalifa, 2013; El-Shater, 1985; Harraz et al., 2012; Mostafa et al., 2023; Wedepohl, 1978). Cu (factor 3, account for 13.21% of the total variance) is negatively correlated with other ions, does appear to be related to a particular bed-rock type that represent the lithological background of the drainage system (Harraz et al., 2012; Redwan & Rammlmair, 2017), and shows a low negative relationship with Cd, Pb, and Zn (− 0.64, − 0.69, and − 0.72 for Co and − 0.21, − 0.24, and − 0.27 for Cu).

**Table 3:** Correlation matrix of the heavy metals of the Wadi Asal sediments. The values in bold denote marked loading. The correlations are significant at *p* < 0.05

Variable	Cd	Cr	Co	Ni	Zn	Pb	Cu	Factor 1	Factor 2	Factor 3
Cd	1.00							**0.78**	− 0.37	− 0.17
Cr	− 0.39	1.00						− 0.78	− **0.56**	− 0.25
Co	− 0.64	0.54	1.00					− 0.86	0.13	0.17
Ni	− 0.34	0.95	0.51	1.00				− 0.72	− **0.55**	− 0.31
Zn	0.53	− 0.45	− 0.72	− 0.43	1.00			**0.80**	− 0.20	− 0.06
Pb	0.83	− 0.32	− 0.69	− 0.25	0.68	1.00		**0.79**	− 0.39	0.00
Cu	− 0.21	0.44	0.21	0.25	− 0.27	− 0.24	1.00	− 0.44	**-0.44**	0.23
Cumulative %								48.89	66.12	79.33

Heavy metal enrichments at some sites away from mine areas, such as E1, E2, E3, E4, E5, and E7 in Wadi Asal, can be attributed to heavy mineral traps and waste transport from the mine. Oxidation, dissolution, and subsequent transport of heavy metals from wastes in Wadi Asal may greatly increase the groundwater’s potential for contamination. In addition, fine particles might be transported by wind over various distances before being deposited in a different ecosystem, and the smallest particle size fraction could be subjected to adsorption–desorption processes on clays and hydrous oxides of Fe and Mn (Abou-El-Anwar & Mekky, 2013; Fernandez & Borrok, 2009; Gantayat et al., 2023; Mileusnić et al., 2014; Redwan & Rammlmair, 2017), or regional contributions from the rocks in the study area. Finally, capillary transport combined with saltwater invasion at sites E01, E02, E03, and E04 in Wadi Asal downstream enhances the dilution of relics of heavy metal carriers and promotes enrichment due to efflorescence that causes Cu, Cd, Pb, Ni, and Zn enrichment (Carmona et al., 2009; Redwan & Rammlmair, 2010).

### Variability and speciation of heavy metals in sediments

**Fig. 5:**
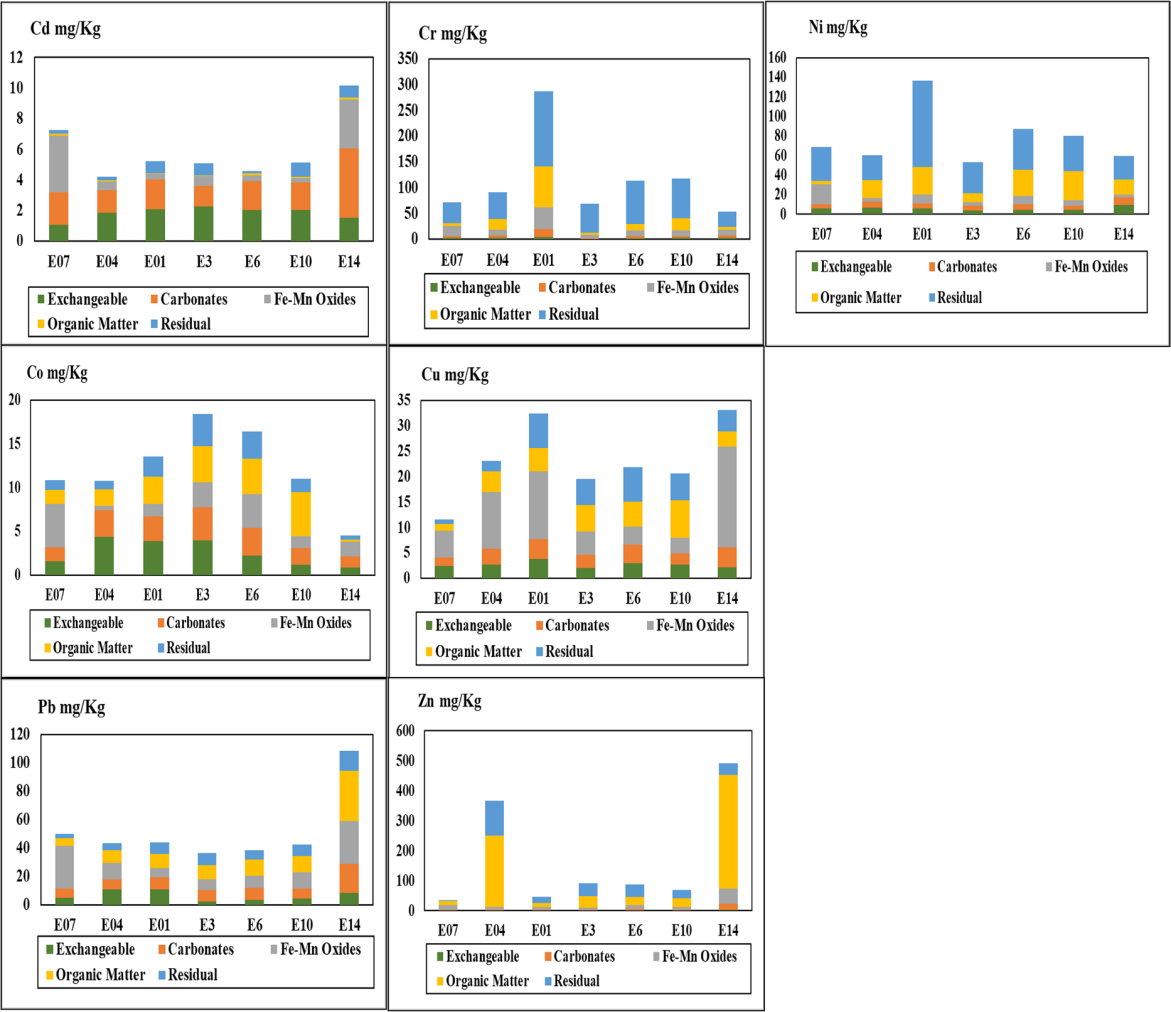
Speciation extraction of heavy metals (Cd, Cu, Pb, Cr, Co, Ni, and Zn) on the selective sediment samples (*n* = 7)

In order to determine the mode of occurrence of heavy metals and their effects on the environment according to their mobility, the Tessier et al. (1979) procedure has been rigorously tested (Howard & Vandenbrink, 1999; Martin et al., 1987) and applied for trace element speciation in sediments (Jone & Turki, 1997; Klink et al., 2019; Li & Thornton, 2001; Li et al., 2001; Ramos et al., 1994, 1999) for the different fractions, exchangeable (F1), carbonate (F2), Fe and Mn oxides (F3), organic (reducible) (F4), and residual fraction (F5) (Fig. 5). Heavy metals mobility and availability in sediments are greatly related to the kinds of binding forms. The decreasing order of mobility and availability of metal forms is in the order: acid soluble (exchangeable & carbonates) forms > reducible forms > oxidizable forms > residual forms (Zimmerman & Weindorf, 2010). In polluted sediments, the heavy metals are generally more mobile, while in unpolluted sediments, they are generally immobile and bind to silicate and primary minerals (Al-Mur, 2020; Sungur et al., 2014).

In the study area, Cd showed high concentrations in bioavailable fractions (F1 + F2 + F3 + F4), ranging from 81.82 to 97.59% compared to its total value, which indicates that the primary anthropogenic source of Cd is from Zn and Pb mining activities. Cd displays vital contents (1.34–4.54 mg/kg, average 2.15 mg/kg) congruently in the carbonate fraction. The elevated values of cadmium in the carbonate fraction are due to its co-precipitation or combination into the crystal lattices of the main phosphate mineral from Wadi Hamadat (north the study area, Khalil & McClay, 2009) (Sengul et al., 2006). Cd exhibits moderate values (1.07–2.26 mg/kg, average 1.83 mg/kg) in the exchangeable fraction, and low values in the residual fraction (more stable) (0.11–0.94 mg/kg, average 0.54 mg/kg). The Fe–Mn oxide fraction has moderate values ranging between 0.30 and 3.72 mg/kg with an average 1.31 mg/kg.

Cu displayed significant values of 69–93.78% in the bioavailable fractions (F1 + F2 + F3 + F4) in the analyzed sediments. The Fe–Mn oxide fraction shows a high level of copper in the study area, accounting for 3.20 to 19.8 mg/kg indicating that a major part of the additional anthropogenic Cu is incorporated in the oxidizable fraction (Adediran & Kramer, 1987; Al-Mur, 2020) (Fig. 5). However, slight Cu increases in the reducible, carbonate, and exchangeable fractions were also reported.

The critical part of Pb occurs in the unstable fractions of Wadi Asal (76.11–94.03%). Very high concentrations of Pb are found in the Fe–Mn oxide and organic matter fractions. The other fractions (exchangeable and carbonates) have moderate mean values, indicating anthropogenic sources of Pb are due to Pb–Zn mining activities. Thus, lead occurs in a free state, depending on its strong mobility under specific environmental conditions (Caporale & Violante, 2016). It is challenging to release Pb from the residual fraction ranging from 5.97 to 23.89% due to its low mobility. The residual fraction (62.93%) in Wadi Asal is the most important fraction responsible for Cr enrichment in sediments. It varied from 29.0 to 146.2 mg/kg by an average of 69.0 mg/kg of the total Cr content, indicating lithogenic provenance due to chromite ores, which is generally a major carrier of Cr in basaltic magmas. Pyroxene and amphibole in basement rocks may be enriched in Cr (Ure & Barrow, 1982; Xia et al., 2023). Thus, it is complicated to release Cr in nature due to its low mobility. The Fe–Mn oxide fraction is the following important species of chromium in Wadi Asal, which has a moderately significant Cr value ranging from 6.48 to 42 with a mean of 16.1 mg/kg, indicating the chemical weathering of chromite minerals. The other fractions are negligible with low Cr concentrations.

The most vital fractions (F1 + F2 + F3 + F4) of Co ranged from 80.15 to 90.48% of the total Co, indicating anthropogenic sources from the Pb–Zn mining activities. These fractions have the highest mobility and can easily release Co into the surrounding environment and the groundwater aquifer (Tessier et al., 1979). The organic matter fraction was the most dominant cobalt binding phase. The residual fraction contains low values of Co varied from 9.52 to 19.85%.

The major content of Ni is hosted in the residual fraction (ranging from 40.16 to 64.52%) of Wadi Asal sediments. It confirms their natural geogenic origin due to the provenance of the Egyptian basement complex rocks (Reimann & de Caritat, 1998; NPI, 2012; Abdel-Rahman et al., 2022). Thus, it is challenging to remove Ni from this fraction. The other fractions (35.48–59.84%) are the available sources of Ni in the environment due to their high mobility, indicating anthropogenic sources of Ni. The highest Zn abundance in the sediments of Wadi Asal is encountered in organic phases, containing (53.46–95.09%) due to Pb–Zn mining activities (Redwan & Rammlmair, 2017). That confirms their ease of release into the environment due to their high mobility.

## Pollution indices and ecological risk assessment of heavy metals

In this study, the contamination factor and risk rating code have been utilized to quantify the presence of heavy metal contamination in sediments close to the Wadi Asal Pb–Zn mine region.

### Contamination factor (CF)

**Fig. 6:**
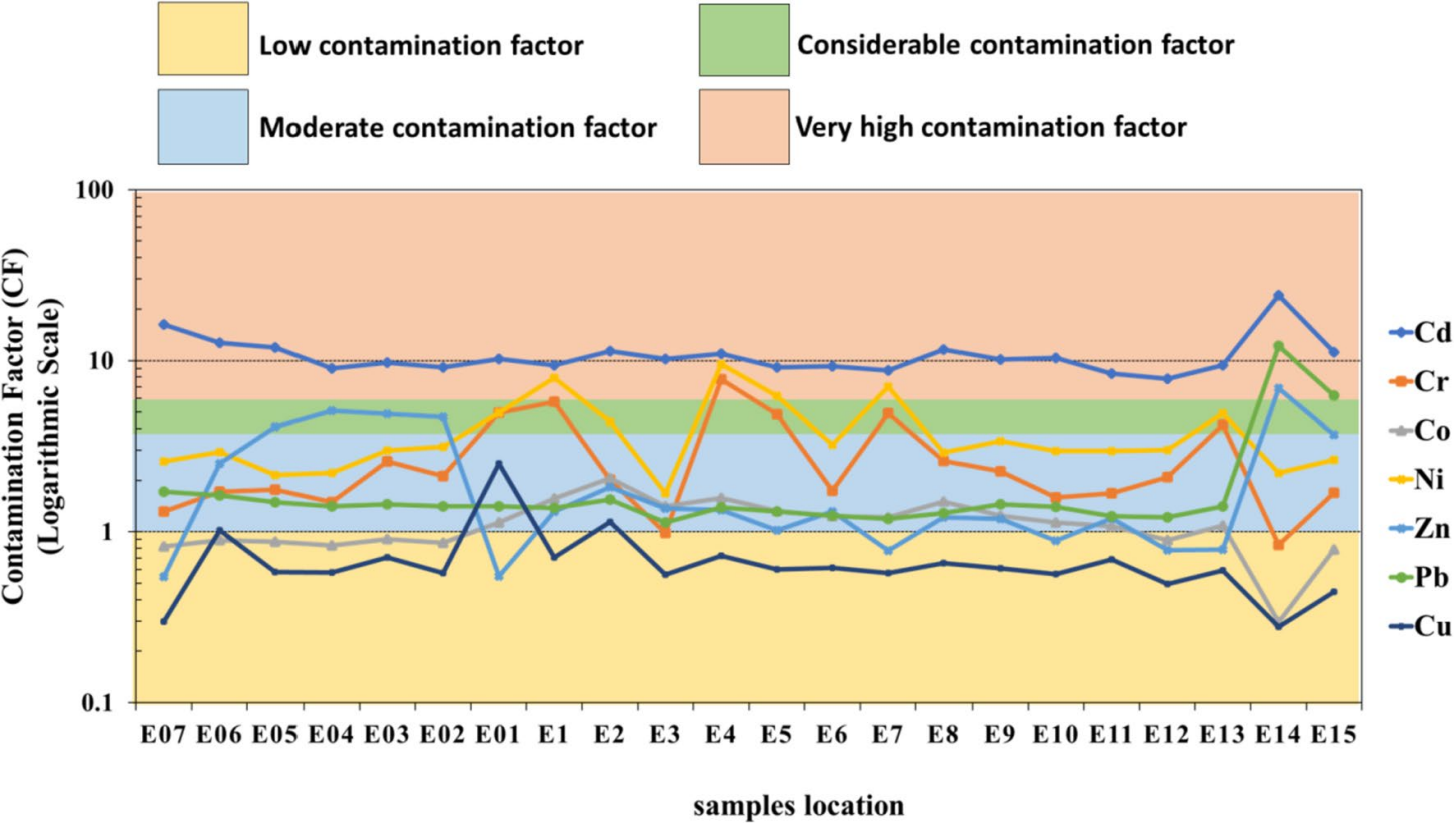
Shows the contamination factor (CF) values in Wadi Asal

According to Hakanson’s (1980) classification, Wadi Asal was very strongly contaminated by Cd, with an average value of 10.96 mg/kg. Ni is considerably polluted, Cr, Co, Pb, and Zn are moderately to strongly polluted, and Cu is lowly polluted in the sediment. The sites E14 and E15 (Asal mine) are very strongly polluted by Cd, Pb, and Zn (Fig. 6). The order of average metal CF is Cd (10.96) > Ni (3.91) > Cr (2.77) > Zn (2.18) > Pb (2.10) > Co (1.12) > Cu (0.70).

### *Geo-accumulation Index (I*_*geo*_*)*

**Fig. 7:**
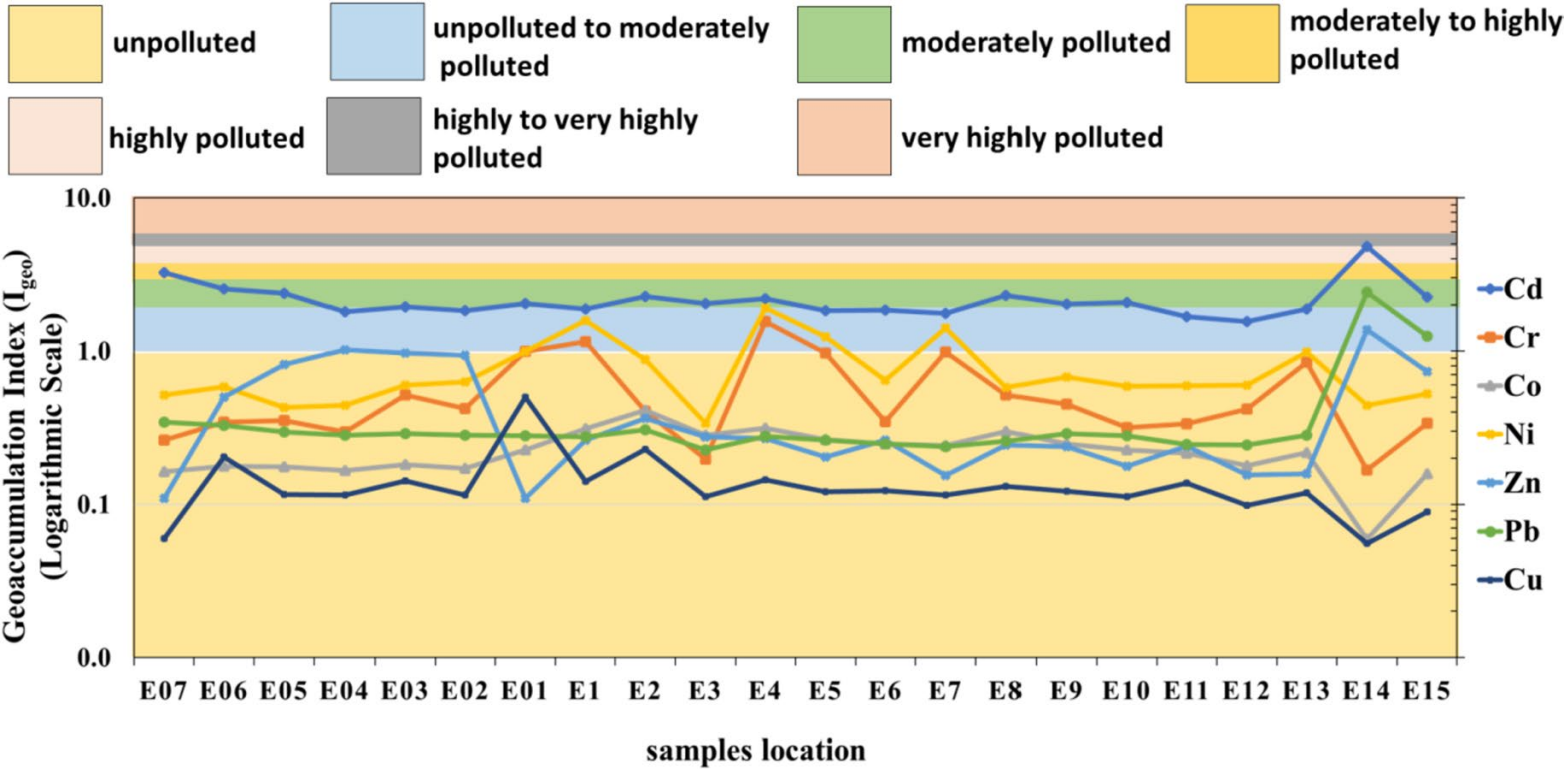
Shows the Geo-accumulation Index (I_geo_) values in Wadi Asal

The resulting I_geo_ values demonstrate that Cd, Pb, and Zn are enriched in the surface sediment in the mine area of Wadi Asal. The Geo-accumulation Index is decreased of average metal in the following order: Cd (2.19) > Ni (0.78) > Cr (0.55) > Zn (0.44) > Pb (0.42) > Co (0.22) > Cu (0.14) in Wadi Asal. The sediments are highly polluted by Cd with a mean value of 2.19, while are moderately polluted by Ni, Cr, Zn, Pb, Co, and Cu. The I_geo_ trends are comparable to the original data. The highest I_geo_ values for Cd, Pb, and Zn occur at sites E14 and E15 near the mine area, while decreases at other sites (Fig. 7). The decrease of I_geo_ indicates that the main source of the enrichments of metals such as Cd, Pb, and Zn in the sediment is the mine site in Wadi Asal.

### Risk assessment code (RAC)

**Fig. 8:**
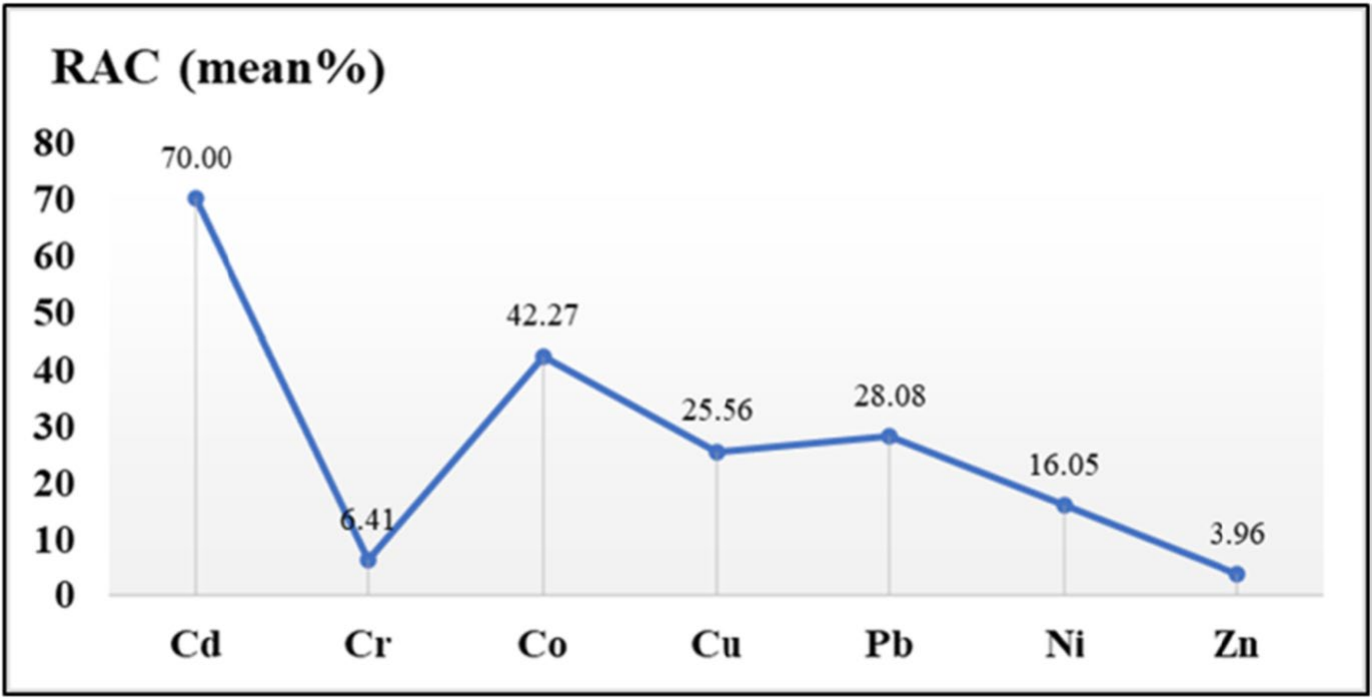
RAC (mean%) values of selected heavy metals in Wadi Asal

The environmental risk of heavy metals (i.e., Cd, Co, Cr, Cu, Ni, Pb, and Zn) was evaluated in the surficial sediments of Wadi Asal by using RAC. The liberated metals from the fractions are classified as weakly bonded. The RAC displayed wide value ranges among the heavy metals. Cd is very mobile and has a very high risk, with a mean of 70% in Wadi Asal. Co is of high risk (42.27%), Pb is of medium risk (28.08%), and Cu is of medium risk (25.56%) in Wadi Asal. Consequently, heavy metals that are assigned to organic matter may become accessible because most of them are water-soluble (Zaky & Abdel-Salam, 2020). RAC of heavy metal values in Wadi Asal sediments follow the order: Cd > Co > Pb > Cu > Ni > Cr > Zn (Fig. 8) from high to moderate risk according to this mobility.

The sediment quality standard provides a way to identify the potential biological influences of sediment (Ma et al., 2015). The threshold effect level (TEL) and the probable effect level (PEL) (Smith et al., 1996) are used to evaluate the ecotoxicology caused by heavy metals in surface sediments in Wadi Asal. The results are shown in Table 4. The median values of the studied heavy metals of Cd, Cr, and Ni were greater than the TEL and the PEL and Pb in the TEL only (Table 4), and adverse effects on living organisms are expected to occur.

**Table 4:** Sediment quality guidelines for metals (i.e., above which negative impacts are likely to occur) and compared to the median values of heavy metals in Wadi Asal

Variable	Median	TEL	PEL
Pb	38.03	35	91.3
Zn	92.15	123	315
Ni	86.83	18	36
Cr	122.10	37.3	90
Cu	23.28	35.7	197
Co	12.55	–	–
Cd	4.18	0.596	3.53

Sediments can absorb a higher level of both toxic and persistent chemicals than water (Al-Mur, 2020). The heavy metal values in the current study showed higher enrichment than other coastal areas of the world, such as the Hurghada area, Red Sea coast, Egypt (Salem et al., 2014), North of Suez Gulf, Egypt (El-Moselhy & Gabal, 2004), Caspian Sea, Iran (Mora & Sheikholeslami, 2002), Black Sea, Turkey (Topcuoglu et al., 2002), Sonora River basin, México (León-García et al., 2022), Al-Hodeidah coast, Yemen (Heba et al., 2004), and Jeddah coastal zone of the Red Sea, Saudi Arabia (Al-Mur, 2020; Al-Mur et al., 2017; Badr et al., 2009).

The results of chemical fractions illustrated that considerable proportions of heavy metals were in the unstable fraction, the toxic metals exert greater potential hazardous influence due to their higher susceptibility, migration capability, and bioavailability with strong anthropogenic sources from the mining activities in the current basin. On the contrary, Cr and Ni exhibited considerably high proportions in residual fraction, demonstrating a rather limited availability in all of the sediment samples. Based on a previous study on the mobility and bioavailability of heavy metals in sediments along the Red Sea, Saudi Arabia, Cu, Zn, and Pb were present in the reducible and oxidizable forms while Cd, Ni, Fe, and Mn tend to accumulate in the carbonate, reducible and organic fractions. The source of heavy metals in the near-shore sediments in the eastern Black Sea might be related to agricultural and industrial activities in the region, especially mining practices. On the other hand, the heavy metal values in the western Black Sea region are influenced by rivers and atmospheric precipitation (Topcuoglu et al., 2002). The Caspian Sea of Iran indicates several metals (As, Cr, Ni) that exhibit concentrations sufficiently high to exceed sediment quality guidelines. Such metals have a high natural background in this mineral-rich region; however, anthropogenic activities, notably mining, may have enhanced the metal burdens in the sediments of the Caspian Sea (Mora & Sheikholeslami, 2002). León-García et al. (2022) recognized that a significant proportion of heavy metals (Cu, Mn, Ni, Pb, and Zn) was associated with the non-residual fraction in the Cananea area, north of the state of Sonora, Mexico, proving these metals have greater mobility and may be bioavailable to living beings due to the presence of the “Buenavista del Cobre” mine, one of the largest copper mines in Mexico.

Enormous volumes of contaminated waste were developed during and after ore extraction and processing. After the mine is closed, these wastes may emit harmful metals or metalloids. The geological map of Wadi Asal (El Aref & Amstutz, 1983; Khalil & McClay, 2009) outlines materials (Pb–Zn) from the mine deserted to the flow path. Most likely, this is the primary source of contamination by heavy metals, chiefly Cd, Pb, and Zn, in Wadi Asal sediments, in addition to the contribution of some hillslope erosion products and finally from downstream tributaries. A mineralogical and chemical background of the drainage paths prior to attaining the mine sites must be anticipated, transport circumstances, groundwater evaporation, and seawater infiltration near the beach. The analyzed samples exhibit regionally significant differences in the pattern of element distribution. Metal distribution in sediment fractions may change as a result of changes in environmental factors such as pH, salinity, redox potential, organic matter, and temperature (Oh et al., 2016). The main factor causing heavy metal enrichment in Wadi Asal is the transport of mine waste and minerals. Some elevated values at some sites away from mine areas may be attributed to mineral traps. Also, due to adsorption–desorption in clays and iron hydroxides, and wind transport of fines (Mileusnić et al., 2014), or regional contributions from the weathered basement rocks, capillary transport and saltwater intrusion at sites E01, E02, E03, and E04 near the beach are the chief sources of Cu, Cd, Pb, Ni, and Zn enrichment (Redwan & Rammlmair, 2010). Without proper mine site rehabilitation, especially along the Red Sea coast, with multiple layers of dry gravel cover and vegetation on top (Redwan & Bamousa, 2019), material movement due to erosion episodes, particularly during high winds and flash flood times, could pose a threat to living organisms through direct consumption or inhalation. Therefore, it is advised to monitor heavy metal levels in fish, water, and beach sediments for future assessment.

## Conclusions

The distribution patterns of heavy metals from the Pb–Zn mine in Wadi Asal, Red Sea, Egypt, were quantified. According to the statistical analysis and aerial distribution maps, the high concentrations of Cd, Cu, Pb, and Zn and moderate concentrations of Cu in the estimated sediments are due to the occurrence of Pb–Zn (Asal mine) mining activities and the surrounding Egyptian basement rocks. The statistical analysis and speciation profiles of Cd in Wadi Asal sediments show that the carbonate fraction of Asal forms the main species of Cd in sediments. The residual fraction is the principal fraction responsible for Cr in sediments, indicating lithogenic provenance. The organic matter fraction was the principal Co-binding phase. The Fe–Mn oxide fraction shows a high concentration of copper. The critical part of Pb occurs in the unstable fractions (Fe–Mn oxide and organic matter). The major content of Ni is hosted in the residual fraction. The most common occurrences of Zn in the sediments are organic phases for Asal. High metal pollution levels were recognized utilizing the contamination factor and risk assessment code in the sediments closest to the mine. The mean metal CF order is Cd > Ni > Cr > Zn > Pb > Co > Cu. The I_geo_ is decreased in the order: Cd > Ni > Cr > Zn > Pb > Co > Cu. Based on the RAC scale, the heavy metal values in sediments are Cd > Co > Pb > Cu > Ni > Cr > Zn. The RAC showed a wide range of values among the heavy metals. For a future assessment, it is advised to carry out seasonal and long-term studies to monitor the amounts of heavy metals in fish, water, and beach sediments to follow the heavy metal accumulations, as well as Pb–Zn mine site restoration techniques around the Red Sea coast and similar areas.

## Data Availability

The datasets generated during and/or analyzed during the current study are available from the corresponding author on reasonable request.
